# Investigation of Production Limits in Manufacturing Microstructured Surfaces Using Micro Coining

**DOI:** 10.3390/mi8110322

**Published:** 2017-10-30

**Authors:** Michael Zahner, Lukas Lentz, Felix Steinlein, Marion Merklein

**Affiliations:** Institute of Manufacturing Technology, Friedrich-Alexander-Universität, Erlangen-Nürnberg, 91058 Erlangen, Germany; lukas.lentz@fau.de (L.L.); felix.steinlein@fau.de (F.S.); marion.merklein@fau.de (M.M.)

**Keywords:** tribology, microstructures, geometrical accuracy

## Abstract

The application of microstructured surfaces is one possible method to reduce friction in lubricated contacts between components with relative movement. Due to this, the energy efficiency and the occurring wear during the operating time of the final products could be decreased. To manufacture structured surfaces economically, a micro coining process was analyzed within this study. This process offers the potential for integration into the established manufacturing processes of different final products, such as tappets used in a valve train. Thus, large-scale production is enabled. To detect the manufacturing limits of the micro coining process, the manufacturing of the coining tools as well as the coining process needs to be investigated. Within this study, the achievable accuracy and the failure of cuboid and cylindrical microstructure elements with selected dimensions were analyzed. For both types of microstructures, the minimal lateral dimensions were detected. Besides the achievable accuracy, correlations between different geometrical dimensions of the micro elements are presented. Additionally, the aspect ratio is detected as the main cause of failure for the micro coining process. In general, the suitability of a coining process for manufacturing microstructured surfaces is proven.

## 1. Introduction

An increasing demand for technical systems with improved performance and reliability, as well as the growing environmental awareness that raw materials are becoming more and more scarce, motivate the need for efficient machine elements. Reducing friction provides an opportunity to increase the energy efficiency of machine elements. Especially in high loaded contacts with relative movement between the contact partners, friction is a major influencing factor on energy losses [1]. A typical contact characterized by these conditions is the cam-tappet-contact [2]. Among other things, the friction and wear in the contact zone of the cam-tappet-contact can be influenced by a modification of component surfaces, for instance by coatings [3] or microstructures [4]. In particular, for the cam-tappet contact, a reduction of friction was proven by Dobrenizki et al. [5] using diamond-like coatings. As a result of additional improvement of wear resistance, for example shown by Lawes et al. in Reference [6], coated tappets are already used in series production [7]. Due to the high costs of coating processes, the microstructuring of functional surfaces is a suitable alternative to improve the tribological conditions in high loaded contacts. Previous investigations have shown that a reduction of friction can be realized by using microstructured surfaces, manufactured by a micro laser ablation process [8]. However, the laser ablation process is not suitable for mass production because of high costs and long single-item production time. Thus, a micro coining process [9] is chosen in order to realize a large-scale production. Such a process can be integrated into the backward extrusion process commonly used for manufacturing tappets and applied in the cam-tapped-contact of the valve train. Based on the fact that already minor geometrical deviations of discrete structure elements lead to significant changes of the tribological conditions [10], the production limits need to be investigated to make use of the full potential of the micro coining process. Within this study, the process limits of tooling and the micro coining process itself are determined.

## 2. Experimental Setup

Within this section, an overview of the used methodology for the manufacturing of microstructured elements is given. Additionally, the materials, machines, and tools employed are presented.

### 2.1. Methodology

**Figure 1:**
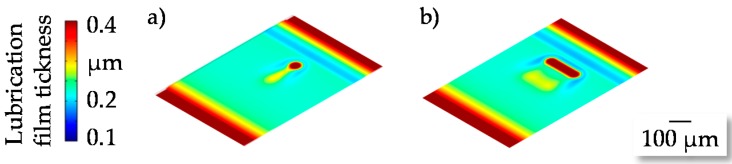
Lubrication film thickness for (**a**) cylindrical; (**b**) cuboid structures adapted from Reference [11].

The investigated manufacturing process of microstructured surfaces is divided into the manufacturing of the coining punch using micro electrical discharge machining (µEDM) and the coining process itself. In the first step, the accuracy and the repeatability of the µEDM process were investigated depending on different geometries and dimensions of the micro pins. Based on these results, the manufactured micro punches were used to generate microstructures. During the micro coining process, the limitations regarding deviations between nominal geometry and generated geometry were additionally investigated. This analysis was necessary since geometrical deviations lead to significant changes in tribological conditions, as shown in Figure 1 by the distribution of lubrication film [11].

**Figure 2:**
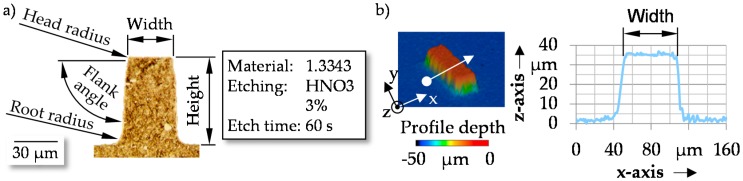
(**a**) Etched cross-section polish through a cuboid micro pin with major pin dimensions and (**b**) example for the measurement of a pin with for a cuboid structure element.

The following explanations concerning the geometrical accuracy and the failure of micro structures refer to cuboid and cylindrical geometries. These types of structures have been identified as appropriate geometries to reduce friction [12]. The width with respect to the diameter was set between 5 µm and 50 µm. Lower values than 5 µm were not investigated, caused by the positioning accuracy of the eroding machine. Structures larger than 50 µm exceed the Hertzian contact width in elasto hydrodynamic contacts like the cam-tappet-contact [12]. Based on this, structures larger than 50 µm are irrelevant for the investigated contact conditions. The pin heights were set between 30 µm and 50 µm. These values exceed previously investigated heights [8]. In particular, an improvement of tribological conditions with increasing height could be shown in Reference [8]. The higher heights lead to taller aspect ratios. This enables the identification of the limits for manufacturing the pins and for the coining process. Additionally, higher pins on the punch enable a targeted height setting using subsequent processes such as grinding. The positions where the pin dimensions were measured are shown in Figure 2a for a cuboid structure element. The real dimensions of the eroded pins and the manufactured structures were analyzed using the laser scanning microscope Keyence VK-X200 (Keyence, Osaka, Japan). The profile of the structures was determined by averaging five cross-sections through the microstructure elements (Figure 2b). To ensure an accurate measurement, a 20× magnification with a step size of 1 µm was chosen.

### 2.2. Materials

For the current study, case hardening steel 1.7131 (Bucher Stahlhandel, Rottweil, Germany) was used as a workpiece material. This is a commonly used material for bucket tappets and other highly stressed machine elements. For the micro coining punch, the high-speed steel 1.3343 (Bartsch Bornheim, Germany) hardened to 62 + 2 HRC (H-O-T, Nuremberg, Germany) was selected as a typical representative in cold forging applications.

### 2.3. Micro Eroding Machine

The micro pins on the coining punch were generated by µEDM. In this study, a Sarix SX-200-HPM (Sarix SA, Sant’Antonino, Switzerland) was used. The system works according to the principle of micro erosion using electrodes made of carbide with a nominal diameter of 288 µm. An integrated wire-cut EDM enables dressing the carbide electrode to the working diameter of 200 µm within a tolerance of ±3 µm. The absolute positioning accuracy of the working area is restricted to ±2 µm for each axis. To prevent thermal influences, the machine works in a temperature-controlled room at 20 °C.

### 2.4. Micro Coining Tool

**Figure 3:**
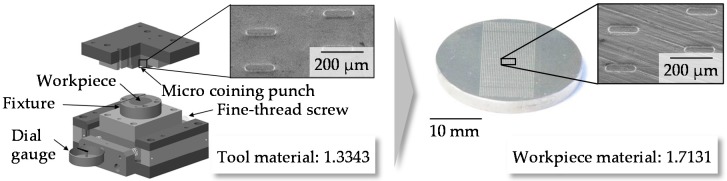
Setup of the micro coining tool and structured workpiece.

Based on the wide range of different microstructure elements with varying dimensions of structure geometries, a coining process (shown in Figure 3) was chosen for the investigations within the study. At the beginning of the coining process, a punch with protruding micro pins moves downwards and is positioned on the workpiece. The workpiece is positioned in a fixture. After reaching a defined coining force, depending on the experimental design, the coining punch moves backwards and the microstructured workpiece can be removed to analyze the structure geometry.

## 3. Results and Discussion

### 3.1. Manufacturing of the Coining Punch

During the manufacturing of the coining punch using µEDM, deviations compared to the nominal pin geometry were determined. These deviations are a result of the axis positioning accuracy of the Sarix SX-200-HPM and the geometry change of the electrode caused by wear. Additionally, the deviation of the nominal electrode diameter caused on the dressing process increases the achievable tolerances. For the determination of the manufacturing limits, the lateral dimensions in terms of width respective of diameter and the flank angle of the pins were investigated. Pin height and edge roundness were not investigated. This is due to the fact that the pin height in particular can be exactly defined using, for example, a subsequent grinding process. With regard to the edge roundness, there was no significant change in the dependence of the important parameters influencing the tribological contact conditions such as the width, diameter, or height of the micro pins.

#### 3.1.1. Pin Width Respective of Diameter

**Figure 4:**
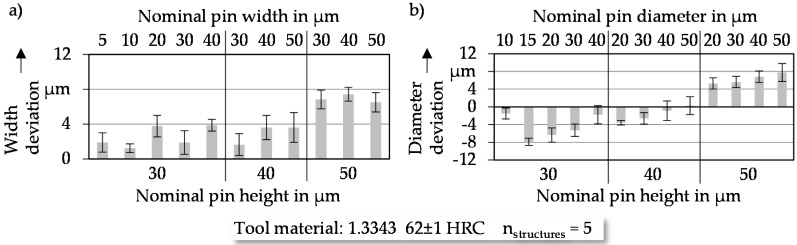
Deviations of lateral dimensions of (**a**) cuboid and (**b**) cylindrical pins.

The absolute width deviations of the cuboid micro pins are shown in Figure 4a. Analyzing the diagram reveals a larger absolute width of structure elements with increasing pin height. The same tendency is shown in Figure 4b for cylindrical elements. Especially for small nominal pin heights, the structures exhibit dimensions smaller than the nominal width. The reliance of width respective of diameter deviation depending on the nominal pin height can be attributed to the used CAM software Esprit 2009.10 (Esprit, Camarillo, CA, USA). With increasing nominal pin height, the time between the infeed of the carbide electrode increases. As a result of the larger infeed time, wear increases and leads to a decreasing electrode diameter. The reduction of electrode diameter is not considered by the CAM software and leads to reduced electric discharge caused by the increased distance between the electrode and the pin. This results in higher deviation values.

Furthermore, the standard deviations in Figure 4 indicate slightly higher deviations for cylindrical structure elements. This is based on the fact that cylindrical pins require the simultaneous moving of two axes in the horizontal plane during the manufacturing process. The cumulated positioning deviation is 41.5% higher than moving just one axis at the same time. This leads to an increase of absolute positioning deviation from ±2.00 µm to ±2.82 µm in the working plane, caused by the axis movement.

If the relative deviations are analyzed, the cuboid structures have values lower than 18.9% referred to the nominal width. The relative deviations of cylindrical structures are lower than 31.8%. Exceptions are detected for a width of 5 µm (22.2%) and a diameter of 15 µm (52.5%). The significant increases of the percentage deviations for these two lateral dimensions are based on the stronger influence of the positioning accuracy. With regard to reproducible micro pin manufacturing, structures smaller than 10 µm for cuboids and 20 µm for cylindrical structures in lateral dimensions are not suitable.

#### 3.1.2. Flank Angle

**Figure 5:**
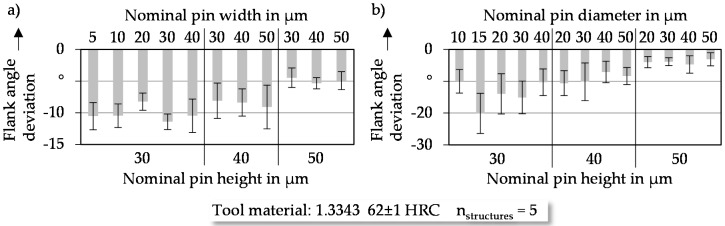
Deviations of flank angle dimensions of (**a**) cuboid and (**b**) cylindrical pins.

In the current study, the nominal flank angle was set to 90°, as manufacturing structures with steep flank angles using micro laser ablation is limited. Both cuboid (Figure 5a) and cylindrical structures (Figure 5b) show a reduction of the averaged flank angle deviation with increasing pin height. A negative deviation means that the flank angle becomes smaller than 90°.

Additionally, the standard deviation becomes smaller with increasing pin height. This tendency is particularly detectable for cylindrical structures in Figure 5b. The standard deviation for a diameter of 30 µm is reduced from 5.15 µm to 1.25 µm, compared to the nominal pin heights of 30 µm and 50 µm. The observed effects may be caused by the electric discharge near the top of the structure elements. With a growing pin height, the material removal at the top of the pins increases until a saturation is reached. This is the case when the distance grows too large for electric discharge. Due to this, the distance between the electrode and the top of the structure element stays nearly constant while the height increases. As a result of this, the deviation of the flank angle decreases for higher pins. Based on these results, the pins have to be tall enough to reach the aspired nominal flank angle. Structure heights in the range of 30 µm and below lead to deviations of up to 20° with high standard deviations. That makes them unsuitable for accurate and reproducible micro pins.

### 3.2. Micro Coining of Structured Surfaces

Besides the manufacturing of microstructured coining punches, the process conditions of the coining process need to be investigated, as deviations from the target geometry of microstructures may influence the tribological conditions in the contact zone of component surfaces. Within the following paragraphs, the changes in width respective of diameter and flank angle for structures without failure during the coining process are analyzed. Based on the failed structures, the limits of manufacturing microstructured surfaces by a coining process are identified in Section 3.3.

#### 3.2.1. Determining of Required Coining Force

**Figure 6:**
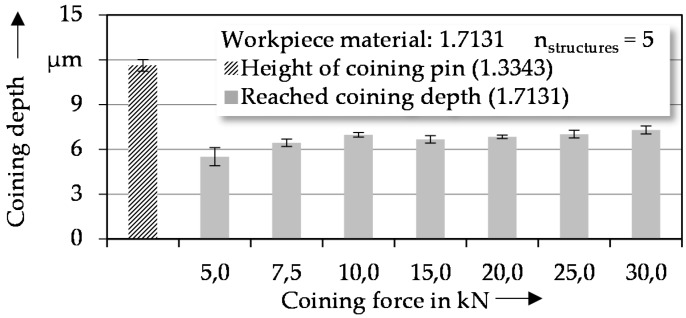
Structure depth in dependence of coining force for cuboid structure elements.

To transfer the geometry of the microstructures on the coining punch onto the workpiece material, a defined coining force is needed. In order to find the minimal required coining force, the structuring process using one microstructured punch was investigated using different coining forces. As reference geometry, cuboid elements with a nominal length, width, and height of 120 µm, 30 µm, and 15 µm, respectively, were chosen. For all investigated coining forces, the reached coining depth is smaller than the measured height of the coining pins caused by the elastic springback of the workpiece material. Below 7.5 kN, as shown in Figure 6, the force is too low to realize a total penetration of the workpiece. Coining forces higher than 7.5 kN show only minor changes of the measured coining depth. These changes can be explained by production-related buckling of the workpieces and inhomogeneities of the workpiece material. The small increase for coining forces up to 25.0 kN and 30.0 kN can be explained by the reduction of roughness peaks, as a result of local stress peaks. To ensure total pin penetration and to minimize the effects of manufacturing influences, the following investigations were carried out using a coining force of 30 kN.

#### 3.2.2. Width Respective of Diameter of Coined Microstructures

**Figure 7:**
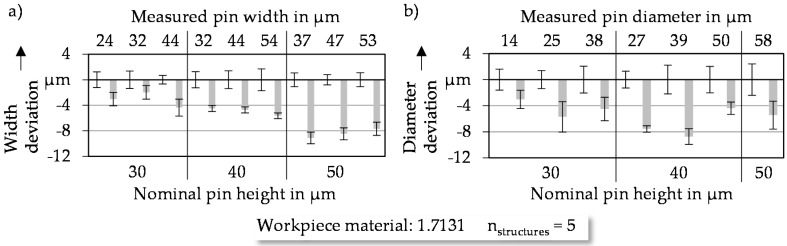
Deviations of lateral dimensions of (**a**) cuboid and (**b**) cylindrical pockets.

In order to define the geometrical limitations of the microstructures caused by the coining process, the geometrical accuracy of the manufactured structures compared to the nominal geometry needs to be identified. This is important in order to achieve efficient structures in terms of the tribological behavior in the contact zone of two components with relative movement. Figure 7a shows the deviations between micro pins of the coining punch and the manufactured cuboid structures. In order to analyze the deviations, the measured pin dimensions are taken as a reference. In addition, the standard deviation arising from the eroding process is supplemented in the diagram left of the measured pin width to evaluate the transferability of the pin geometry on the workpiece by the coining process. If the standard deviation stays nearly the same after the coining process, the deviations are considered to be some kind of systematic characteristic. Both cuboid (Figure 7a) and cylindrical (Figure 7b) pockets show a reduction of width respective of diameter compared to the micro coining pin, caused by elastic springback after removing the coining punch. The results of Figure 7a lead to the conclusion that taller pin heights lead to larger width deviations. Analyzing the cylindrical pockets in Figure 7b does not confirm this tendency.

To explain the width deviations, the flank angles of the micro pins on the coining punch need to be analyzed. As seen in Figure 5, taller pins usually lead to higher flank angles close to 90°. This results in a springback parallel to the surface of the workpiece. With decreasing flank angles, the amount of the springback parallel to the workpiece surface and thus the measured deviation decreases. In the case of cylindrical structures, the pin dimensions are already smaller than the nominal geometry based on the effect of cumulated positioning deviation. Additionally, the elastic springback leads to a further reduction of the diameter of the formed pockets. For cuboid structures the deviations during the manufacturing of the coining punch and the elastic springback are nearly equal. Comparing the standard deviations of the microstructures before and after the coining process by means of width respective of diameter indicates that the standard deviation stays nearly constant. Small sporadic deviations could be justified by the measuring of the structure elements, as it could not be ensured that measurements were always taken at the dead even position.

#### 3.2.3. Flank Angle of Coined Microstructures

**Figure 8:**
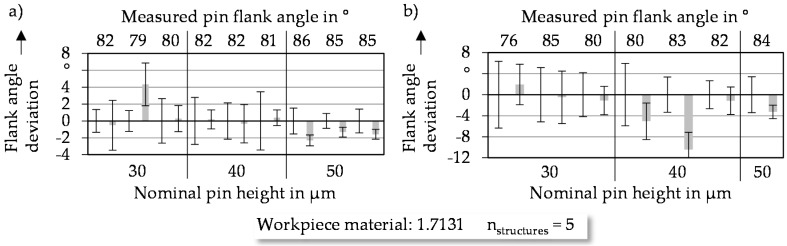
Deviations of flank angle dimensions of (**a**) cuboid and (**b**) cylindrical pockets.

To ensure the dependency between the flank angle and the measured pin width mentioned in Section 3.2.1, the accurate transfer of the flank angle from the micro pin through the workpiece using a micro coining process has to be verified. The measured flank angle for each coined micro pocket is displayed for cuboid structures in Figure 8a, as well as for cylindrical structures in Figure 8b. Especially for the cuboid structures, the flank angle of coined pockets is nearly identical to the coining pins, except for one outlier with a measured flank angle of 79° and a nominal pin height of 30 µm. A similar behavior investigating cylindrical structures leads to the conclusion that the flank angle can be transferred without minimal deviations by a coining process and that the elastic springback occurs orthogonal to the surface of the structure elements.

### 3.3. Failure Limits Using Micro Coining

**Figure 9:**
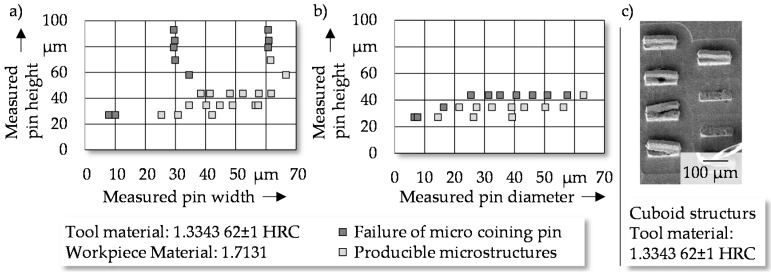
Failure of micro coining pins in dependence of the aspect ratio for (**a**) cuboid and (**b**) cylindrical structure elements, as well as (**c**) image of failed cuboid pins.

Besides the geometrical accuracy of the manufactured structures compared to the nominal geometry, the technical failure of the pins needs to be investigated. The technical failure was defined by the cracking of the micro pins on the coining punch. In order to define the geometrical limitations of the microstructures, the aspect ratio was identified as the critical criterion for pin failure. Based on this, the aspect ratio for each investigated cuboid (Figure 9a) and cylindrical (Figure 9b) structure element was determined by the measured dimensions. The aspect ratio is calculated as the quotient of pin height divided by the width respective of the diameter of the pin.

After coining 30 times, technical pin failure was observed for cuboid elements with an aspect ratio higher than 1.13. Cylindrical pins allowed aspect ratios up to 1.60 without failure. The reduced possible aspect ratio of cuboid structures can be explained by a larger cross-section caused by the cuboid length of 120 µm. The larger cross-section requires a higher material displacement of the workpiece material, which induces higher axial forces and leads to buckling or shearing during the penetration of the pin (Figure 9c). The initial damage results in a tearing off of the micro pin during the return stroke, caused by the induced tensile stress.

## 4. Conclusions and Outlook

In this article the process limits of manufacturing coining punches by μEDM were determined. It has been shown that it is possible to manufacture micro pins accurately and reproducibly within the required dimensions, given by the conditions in the cam-tappet-contact. For the coining process itself, the aspect ratio was identified as a mechanical failure criterion of the micro pins. The dimensions of the manufactured structures in the workpiece material are smaller compared to the pin geometry on the punch. This is due to the elastic springback. For the resulting structure width as a result of the elastic springback, a dependence of the flank angle was identified. Further investigations will focus on the characterization of edge rounding and targeted flank angles departing from 90°, as well as the influence of lubrication and holding time during the coining process. In addition, the integration of the micro coining process into an extrusion process used to manufacture tappets should be investigated to identify interactions between both processes and to analyze the effects on the geometrical accuracy of the micro structure elements.
